# Development of a Web-Based, Guided Self-help, Acceptance and Commitment Therapy–Based Intervention for Weight Loss Maintenance: Evidence-, Theory-, and Person-Based Approach

**DOI:** 10.2196/31801

**Published:** 2022-01-07

**Authors:** Rebecca Richards, Rebecca A Jones, Fiona Whittle, Carly A Hughes, Andrew J Hill, Emma R Lawlor, Jennifer Bostock, Sarah Bates, Penny R Breeze, Alan Brennan, Chloe V Thomas, Marie Stubbings, Jennifer Woolston, Simon J Griffin, Amy L Ahern

**Affiliations:** 1 Medical Research Council Epidemiology Unit University of Cambridge School of Clinical Medicine Cambridge United Kingdom; 2 Fakenham Medical Practice Fakenham United Kingdom; 3 School of Medicine University of Leeds Leeds United Kingdom; 4 Patient and Public Involvement Representative Kent United Kingdom; 5 School of Health and Related Research The University of Sheffield Sheffield United Kingdom

**Keywords:** weight management, eHealth, acceptance and commitment therapy, third-wave cognitive behavioral therapy, guided self-help

## Abstract

**Background:**

The long-term impact and cost-effectiveness of weight management programs depend on posttreatment weight maintenance. There is growing evidence that interventions based on third-wave cognitive behavioral therapy, particularly acceptance and commitment therapy (ACT), could improve long-term weight management; however, these interventions are typically delivered face-to-face by psychologists, which limits the scalability of these types of intervention.

**Objective:**

The aim of this study is to use an evidence-, theory-, and person-based approach to develop an ACT-based intervention for weight loss maintenance that uses digital technology and nonspecialist guidance to minimize the resources needed for delivery at scale.

**Methods:**

Intervention development was guided by the Medical Research Council framework for the development of complex interventions in health care, Intervention Mapping Protocol, and a person-based approach for enhancing the acceptability and feasibility of interventions. Work was conducted in two phases: phase 1 consisted of collating and analyzing existing and new primary evidence and phase 2 consisted of theoretical modeling and intervention development. Phase 1 included a synthesis of existing evidence on weight loss maintenance from previous research, a systematic review and network meta-analysis of third-wave cognitive behavioral therapy interventions for weight management, a qualitative interview study of experiences of weight loss maintenance, and the modeling of a justifiable cost for a weight loss maintenance program. Phase 2 included the iterative development of guiding principles, a logic model, and the intervention design and content. Target user and stakeholder panels were established to inform each phase of development, and user testing of successive iterations of the prototype intervention was conducted.

**Results:**

This process resulted in a guided self-help ACT-based intervention called SWiM (Supporting Weight Management). SWiM is a 4-month program consisting of weekly web-based sessions for 13 consecutive weeks followed by a 4-week break for participants to reflect and practice their new skills and a final session at week 18. Each session consists of psychoeducational content, reflective exercises, and behavioral experiments. SWiM includes specific sessions on key determinants of weight loss maintenance, including developing skills to manage high-risk situations for lapses, creating new helpful habits, breaking old unhelpful habits, and learning to manage interpersonal relationships and their impact on weight management. A trained, nonspecialist coach provides guidance for the participants through the program with 4 scheduled 30-minute telephone calls and 3 further optional calls.

**Conclusions:**

This comprehensive approach facilitated the development of an intervention that is based on scientific theory and evidence for supporting people with weight loss maintenance and is grounded in the experiences of the target users and the context in which it is intended to be delivered. The intervention will be refined based on the findings of a planned pilot randomized controlled trial.

## Introduction

### Background

About 33% of UK adults are overweight, and a further 28% live with obesity [1]. Behavioral weight management programs (BWMPs) are the most commonly used treatment for overweight and obesity and typically use behavior change techniques (BCTs), such as self-monitoring, goal setting, stimulus control, and social support, to facilitate energy restriction and increase physical activity [2]. BWMPs can support initial weight loss of approximately 5% to 10% of body weight and are associated with improvements in the risk of diabetes, cardiovascular diseases, and related metabolic disorders [3,4]. However, systematic reviews have shown that even after a gold standard specialist–led BWMPs, most individuals regain weight within 3-5 years [5,6]. The cost-effectiveness and long-term health impact of these programs depend on the maintenance of posttreatment weight loss [7]. Although the extended use of traditional behavioral strategies can improve weight loss maintenance to some extent [8], alternative approaches are needed to better support weight loss maintenance and maximize the long-term health benefits of BWMPs.

There is growing evidence that third-wave cognitive behavioral therapies (3wCBTs) have better long-term outcomes for weight management than standard behavioral therapy (SBT) [9] and thus may be a more effective approach to support weight loss maintenance. The term 3wCBT refers to a set of behavioral and cognitive approaches that focus on a person’s *relationship* with their thoughts, rather than the *content*, as in traditional cognitive behavioral therapy, and centers on concepts such as mindfulness, acceptance, values, and goals, among others [10]. Such approaches include acceptance and commitment therapy (ACT), mindfulness-based cognitive therapy, compassion-focused therapy, and dialectical behavioral therapy. These approaches encourage the acceptance and tolerance of aversive internal experiences (eg, food cravings and physical discomfort) using strategies such as present-moment awareness and cognitive defusion. In relation to weight management, it is hypothesized that developing these skills facilitates improved recognition of internal and external cues to overeat and behavioral responses that move a person toward their value-based goals [10]. In addition, 3wCBT encourages compassion toward the self, which may help prevent discouragement following minor lapses. Systematic reviews have also shown that 3wCBT interventions can improve psychological determinants of weight loss maintenance that have been identified in previous qualitative and quantitative studies, such as self-regulation, autonomous motivation, dietary restraint, disinhibition, negative mood, and emotional eating [9,11-13]. Despite growing evidence that shows that 3wCBT interventions may improve long-term weight management, there is uncertainty regarding their scalability and affordability. These interventions are usually psychologist-led, and psychologists specializing in obesity are scarce and costly. Following the success of cognitive behavioral therapy delivered using technology and trained nonspecialists [14,15], recent, early-phase studies have shown that 3wCBT for weight management can also be delivered remotely, with findings highlighting improvements in weight management and determinants of weight loss maintenance, including experiential avoidance, psychological flexibility, and binge eating [16-18]. Furthermore, a systematic review of 3wCBT eHealth interventions to improve mental health outcomes reported that these programs were feasible and acceptable to participants in practice [19].

### Objectives

This study aims to develop an ACT-based intervention to be delivered using digital technology and nonspecialists to support adults with overweight and obesity to maintain their weight loss following completion of a BWMP. To develop a relevant, engaging, and effective intervention, careful planning and a design process are required, particularly when translating health care interventions into a digital format [20]. Such a process facilitates the development of interventions that are designed to address the identified needs of the target users which are grounded in their experiences, and provides an opportunity to minimize potential barriers to successful implementation. This paper reports the evidence-, theory-, and person-based approach that was used to develop this intervention and the findings of this approach.

## Methods

### Overview

We used a systematic and iterative intervention development process guided by 3 frameworks: the Medical Research Council (MRC) framework for the development of complex interventions in health care [21], the Intervention Mapping Protocol (IMP) [22], and a person-based approach for enhancing the acceptability and feasibility of interventions [23]. The MRC framework encourages a 4-phase approach including development, feasibility and piloting, evaluation, and implementation, with each phase requiring a body of work to be conducted [21]. Similarly, the IMP sets out 6 steps for intervention development, which broadly include the consideration of the target behavior from an ecological perspective, the participation of stakeholders in all phases of development, and the integrated use of theory and evidence [22]. Finally, a person-based approach, which advocates for in-depth exploration of the target users and their context, was used to complement these frameworks to facilitate the development of a relevant and engaging intervention [23].

In line with these frameworks, we conducted an initial needs assessment through workshops and focus groups with a panel of key stakeholders (including local commissioners and providers of weight management and diabetes services in the United Kingdom National Health Service [NHS] and local authority) and a panel of target user representatives (16 adults who had lost weight and attempted weight loss maintenance and were independent of the research study, 9 of whom regularly responded and attended meetings). Workshops and focus groups took place before the first COVID-19 lockdown in the United Kingdom. Both panels expressed a need for a weight loss maintenance program to support adults who have completed a BWMP (including NHS, local authority and commercial weight management, and diabetes prevention programs) within the last 3 months. Adults who use insulin, have undergone bariatric surgery in the past 2 years or have planned a surgery, are pregnant or planning a pregnancy, or have a current diagnosis of an eating disorder will not be eligible for this weight loss maintenance program based on expert stakeholders advise that the specific support needs of these groups are beyond the remit of this intervention. The stakeholder panel reported that there were insufficient resources for a program to be psychologist-led and thus required a program that could be delivered to a large number of adults from the target population at low cost. The locality of these meetings (East Anglia) has both rural areas and poor transport links. Accordingly, the target user and stakeholder panels favored a remotely delivered intervention comprising a web-based platform with telephone support.

**Figure 1:**
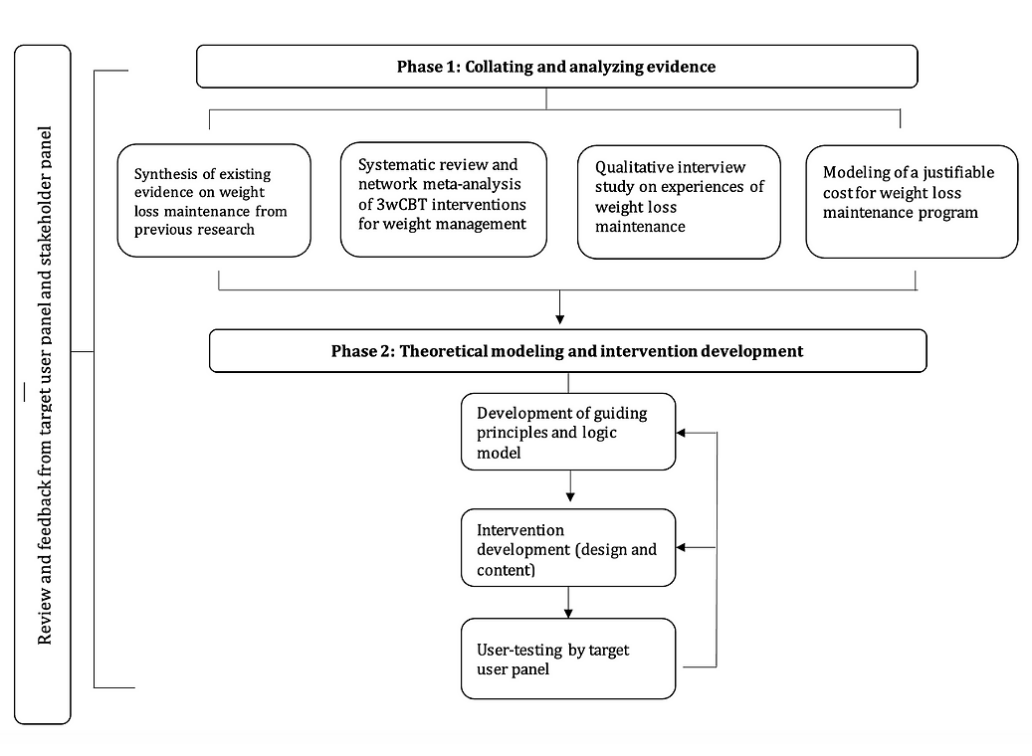
Flowchart outlining each phase of work. 3wCBT: third-wave cognitive behavioral therapies.

We conducted two phases of work to plan and design the intervention: phase 1 consisted of collating and analyzing existing and new primary evidence, and phase 2 consisted of theoretical modeling and iterative intervention development, including repeated cycles of user testing and refinement (Figure 1). The target user and stakeholder panels were involved in each of the work phases to maximize the feasibility and acceptability of the intervention from the perspectives of the target users, practitioners, and service commissioners. The members of the research team regularly met with both panels. The patient and public involvement representative on the research team (JB) chaired the target user panel meetings to facilitate a dynamic and informal environment, whereby members felt empowered to provide critical feedback. Findings and materials from both phases of work, including the theoretical modeling and intervention content, were presented to each panel at key stages of development for review and feedback. Panel members who could not attend meetings were sent materials via email and were able to provide feedback via email or telephone. Feedback was discussed with the research team and incorporated into revised materials, where appropriate. During each panel meeting, we discussed any changes we had made to the intervention, and if changes recommended by the panels had not been implemented, we discussed the reasons for this.

### Phase 1: Collating and Analyzing Evidence

#### Overview

We collated evidence on weight loss maintenance from previous research, including a systematic review and synthesis of qualitative studies of weight loss maintenance [24] and systematic reviews of theories of behavior-change maintenance [25], the determinants of weight loss maintenance [26], and the effectiveness of weight loss maintenance interventions [27], as well as our knowledge of the wider literature. In addition, we conducted primary research to fill several evidence gaps that we identified. Full reports of each of these primary studies have been published elsewhere [9,28]. The key methods and findings relevant to the intervention development are briefly described below.

#### Systematic Review and Network Meta-analysis of Evidence on 3wCBT-Based Interventions for Weight Management

##### Purpose

Previous systematic reviews of 3wCBT interventions were not comprehensive and had several methodological limitations. We conducted a systematic review and network meta-analysis of evidence on 3wCBT interventions for weight management to examine the relative effectiveness of different approaches (eg, ACT vs mindfulness-based cognitive therapy) and to identify whether specific intervention characteristics were associated with better outcomes.

##### Methods

This systematic review and network meta-analysis evaluated the effects of 3wCBT for weight management on body weight and psychological and physical health outcomes in adults with overweight and obesity [9]. A total of 21 randomized controlled trials that included participants with a BMI ≥25 kg/m^2^, a 3wCBT intervention for weight management, and measurement of body weight or BMI before intervention and ≥3 months after baseline were identified through database searches and included in the meta-analyses. Pairwise random-effects meta-analysis compared 3wCBT with SBT or no or minimal intervention. A network meta-analysis was conducted to investigate whether specific types of 3wCBT were more effective than others. Meta-regression was conducted to identify whether specific intervention characteristics were associated with better outcomes.

##### Results

We found moderate to high quality evidence suggesting that 3wCBT interventions result in greater weight loss compared with SBT and no or minimal interventions after intervention and at the 12- and 24-month follow-up. The network meta-analysis found that ACT-based interventions had the most consistent evidence of effectiveness, indicating greater weight loss compared with SBT after intervention and at the 12- and 24-month follow-up. ACT was ranked as the best intervention for up to 12 months and was the only 3wCBT to report weight outcomes at 24 and 36 months. Meta-regression did not identify any specific intervention characteristics associated with better outcomes. The overall findings of this review support our decision to develop an ACT-based intervention to support the long-term maintenance of weight loss. The findings of our review also confirmed that to date, there is no available evidence on the effectiveness of ACT-based interventions that are designed to support weight loss maintenance and delivered remotely using digital technology and nonspecialists.

#### Qualitative Interview Study on Experiences of Weight Loss Maintenance

##### Purpose

A recent systematic review and qualitative synthesis identified a dearth of qualitative studies that directly compared the experiences of people who had maintained their weight loss following a BWMP with the experiences of those who had regained the weight [24]. No studies have explored the experiences of weight loss maintenance beyond 1 year. We conducted a qualitative study to identify cognitive and behavioral strategies used to overcome lapses and prevent relapse among weight loss *maintainers* and *regainers*, who had lost weight several years previously.

##### Methods

We conducted semistructured interviews [28] with 26 participants (15/26, 58% female) from the Weight loss program Referrals for Adults in Primary care (WRAP) trial [8]. Participants randomized to the intervention arms of the WRAP trial (referral to 12 or 52 weeks of a commercial weight loss program) and who had lost ≥5% baseline weight during the active intervention (year 0-1) were recruited at the 5-year follow-up. Interview participants were purposively sampled for a split of postintervention weight trajectories (maintainers vs regainers) and a range of demographic characteristics. Interview questions focused on postprogram experiences, including identifying the cognitive and behavioral strategies employed in efforts to maintain weight loss over time. Thematic analyses explored the differences in experiences of weight loss maintenance between participants who had maintained their weight over 5 years and those who had regained weight.

##### Results

Maintainers reported using more self-regulation techniques (eg, self-monitoring and planning), anticipated lapses (particularly for social occasions), and made conscious plans to compensate for lapses and continue to manage their weight. In contrast, although regainers made some efforts to self-regulate their behavior, they did not tend to make plans to avoid or overcome lapses, used relaxed dietary monitoring, and had particular difficulty navigating the role of food within interpersonal relationships. The findings of this study highlighted that behavioral monitoring, planning, and managing interpersonal relationships were important skills for overcoming lapses and preventing relapse after participating in a weight management program. This study suggests that creating a weight maintenance plan and making specific plans for high-risk situations should be a key focus of our weight loss maintenance intervention. In addition, the findings highlighted that skills in managing interpersonal relationships with regard to food should also be included.

#### Modeling of a Justifiable Cost for Weight Loss Maintenance Program

##### Purpose

In the absence of data on the cost-effectiveness of the proposed intervention, we modeled the maximum justifiable cost of a weight loss maintenance intervention, given an estimated intervention effect and a specified incremental cost-effectiveness ratio (ICER).

##### Methods

We estimated the initial weight loss and the difference between intervention and control at 12 months by meta-analyzing data from previous studies of behavioral weight loss maintenance interventions identified from 2 recent systematic reviews [27,29]. We then used the School for Public Health Research Diabetes prevention model [30] to estimate the long-term quality-adjusted life year (QALY) gains and health care costs associated with this intervention effect in 1) individuals with a BMI of ≥28 kg/m^2^ without diabetes, and 2) individuals with a diagnosis of type 2 diabetes. A sensitivity analysis was conducted around the rate of regain, duration of effect, and initial weight loss. We set the ICER at £20,000 (US $26,477.30) per QALY (UK NHS benchmark).

##### Results

The estimated intervention effect from the meta-analysis was a 1.5 kg difference at 12 months. The justifiable cost for an intervention achieving this effect at an ICER of £20,000 (US $26,477.30) per QALY varied from £29.98 (US $39.69) to £203.77 (US $269.76). We set a budget for our intervention of approximately £100 (US $132.39).

### Phase 2: Theoretical Modeling and Intervention Development

#### Guiding Principles and Logic Model

##### Purpose

To guide the intervention development, we developed a logic model and guiding principles to summarize how the intervention would support behavior change.

##### Methods

Following the person-based approach [23]*,* we developed a set of guiding principles to specify the intervention design objectives (what the intervention must do to meet the needs of the target user and enhance engagement with the intervention) and the intervention features required to achieve the design objectives in practice. In keeping with the IMP [22], we developed a logic model to describe the hypothesized mechanisms of change. We used the evidence collated in phase 1 to develop the initial logic model and guiding principles. These were presented to the target user and stakeholder panels at each meeting and revised accordingly to incorporate feedback and any additional needs that were identified during the development process.

##### Results

**Figure 2:**
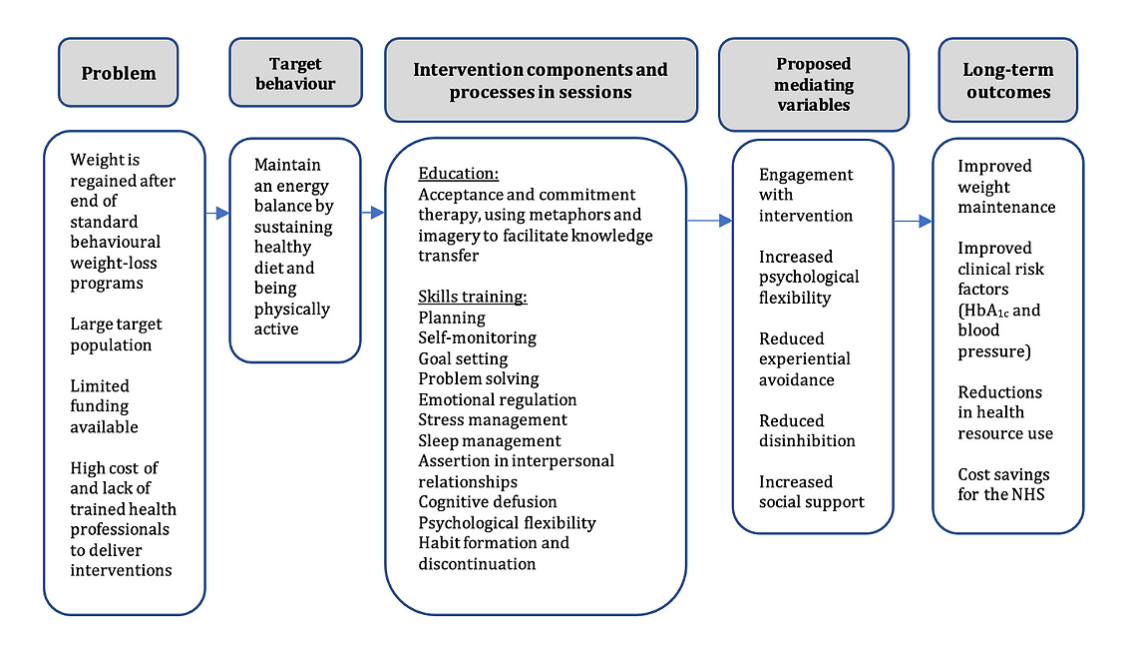
Logic model. HbA_1c_: glycated hemoglobin A_1c_; NHS: National Health Service.

The key intervention objectives and design features of the guiding principles and supporting evidence are listed in Multimedia Appendix 1 [9,14,15,19,24,28,31]. The logic model of the hypothesized mechanisms of change in the intervention is presented in Figure 2. The intervention objectives identified from synthesized quantitative and qualitative primary evidence, existing literature, and input from the target user and stakeholder panels included the following:

1. to deliver an effective ACT-based intervention that can be delivered remotely and at scale at a cost of approximately £100 (US $132.39) per participant;
2. to build on the participants’ existing knowledge and experience of weight management and what works for them;
3. to encourage participants to take ownership of their weight management in the long term;
4. to support participants in planning to navigate factors that commonly derail weight loss maintenance, such as high-risk social situations and old unhelpful habits.

#### Intervention Development (Design and Content)

##### Purpose

The purpose was to develop a prototype web-based, guided self-help, ACT-based intervention, including format, content, design, and function.

##### Methods

We used the guiding principles (Multimedia Appendix 1) to develop an outline of the intervention structure and then worked with a team of software developers (Cauldron Inc) to design the wireframe of the web-based platform. Each stage was reviewed by the target user and stakeholder panels, and feedback was incorporated. The core content was ACT-based; publicly available treatment protocols, behavioral experiments, and reflective exercises were obtained for ACT-based weight management interventions and more generic ACT-based interventions [11,17,18,32-35] and reviewed in the light of our guiding principles and evidence syntheses to identify key skills and strategies to include. SBT behaviour change techniques (eg, goal setting and planning) were also considered for inclusion when these were supported by our phase 1 work and could be implemented in a manner consistent with an ACT-based approach. We then drafted the intervention content materials, which were reviewed by the target user and stakeholder panels and revised accordingly. We worked with the target user panel and a graphic designer to create images to illustrate important concepts and metaphors from ACT. We chose a cartoon style with bold colors that were engaging and added a light-hearted touch to the content. The images were reviewed by the research team and target user panel, and refined accordingly. The software developers created a wireframe for the intervention platform and released an alpha version to which we added the content.

The prototype of the website platform was tested using multiple rounds of think-aloud protocols in line with the person-based approach [23]. Common themes in user experience were identified, and a focus group was held with the target user panel, software developers, and research team to discuss and agree on what could or should be changed. Examples of feedback and changes are listed in Table 1. The proposed changes were reviewed to determine the extent to which they were important for behavior change, consistent with the guiding principles, uncontroversial, technically feasible to implement, and repeated by more than one person [36]. A beta version of the website platform was then released for a wider group of target users to test remotely. Further feedback was incorporated into the final version of the platform, which will be used in a planned feasibility trial. Semistructured scripts for the coach telephone calls and a coach training manual were developed by the research team.

The Behavior Change Wheel, COM-B model, Theoretical Domains Framework (TDF), and behavior change taxonomy [37] were used to code the theoretical determinants, intervention functions, and BCTs of the intervention. This behavioral analysis was conducted using the full intervention content and coach training manual. Specifying interventions using this formal and reliable classification of behavior-change components facilitates the evaluation of behavior-change interventions and their mechanisms of action. In addition, coding for BCTs facilitates the comparison among behavior change interventions and evaluation of technique efficacy in systematic reviews [38-40].

**Table 1:** Examples of feedback from the target user panel through the development process and changes that we made.

Target user feedback on intervention components		Changes we made
**Content**		
	Want more focus on action with instructions and less on theory.	Some theory is important for this intervention; however, we altered the balance of text and theory and highlighted action-oriented content within the pages.
	Some of the language and terminology is difficult to understand.	We revised the wording of session content to use more lay terms and added clear examples to facilitate understanding, and images were used throughout to illustrate abstract ideas.
	Users found the concept of values difficult to understand, and some felt that it could have moral or pejorative undertones.	The session on values was rewritten based on target user feedback, and we worked with the target user panel to find ways of describing the concept of values in a more salient and acceptable way.
	Sessions contain too much block text; users want more color and visuals to break this up.	We worked with a graphic designer to create over 30 illustrations to help break large sessions and illustrate key metaphors and learning points.
**Presentation**		
	Some blocks of text are too long.	We edited all content to be more concise and increased the number of pages within a session so that there were fewer words on each page.
	Too much white space makes the session pages feel dull. The text should be centered, as it can be difficult to read if the text runs across the screen.	The background artwork from the home page was copied across to form a border around a smaller content box, centered on the page.
	Referring to Chris the Supporting Weight Management character with gender neutral pronouns sounds unusual and the text sounds grammatically incorrect.	We revised the text so that it referred to Chris as male. Future iterations of Supporting Weight Management may have different options for the character, including its name and pronouns.
	Font size is too small, and some participants (particularly those in the older age group) may struggle to read the text.	The standard font size was increased across the platform. The website was built dynamically to enable browser level zoom function without distorting the page view.
**Function**		
	When a session starts, it would be good to have an idea of how long it takes to complete it and an indicator of how far you are through the session.	A progress bar was included at the top of each page to indicate each progress through the session.
	When tables (or other exercises) are populated with data, this is not reflected when the same data are used later on in other exercises.	When data are entered into tables (or exercises), this automatically populates other corresponding tables (or exercises) that appear later on.

#### Intervention

The outline of the final intervention structure is shown in Multimedia Appendix 2. To achieve the intervention objectives, a guided self-help, ACT-based intervention was created, which will be delivered remotely via a web-based platform. We named the intervention SWiM (Supporting Weight Management). The ethos of SWiM is to use ACT-based skills and strategies to help people with overweight and obesity who have lost weight to reflect on what has worked (and not worked) in the past, build on what works for them, and learn new strategies to overcome challenges that typically derail weight loss maintenance. Multimedia Appendix 3 outlines how the intervention components addressed each of the core ACT processes.

To enhance engagement and ensure understanding of the intervention content, participants will receive telephone support from a coach. To ensure that the intervention costs remain within the justifiable costs calculated (estimated cost of approximately £100 [US $132.39] per participant) and emulate delivery in a pragmatic setting, nonspecialists will be recruited and trained for this role and will work remotely across a large geographic area, effectively using a *call center* approach. The intervention will start by encouraging participants to reflect on their previous experiences and develop a personalized weight loss maintenance plan. SWiM is diet agnostic, which means that participants may follow a diet that is consistent with their previous positive experiences of weight management. SWiM is a 4-month program, which consists of weekly web-based *SWiM Sessions* for the first 13 weeks, followed by a 4-week break for reflection and practicing of new skills, with a final session at week 18. Each session consists of psychoeducational content, reflective exercises, and behavioral experiments. Between sessions, participants are asked to complete further reflective exercises and behavioral experiments, called *SWiM Practice*. The program content will include specific sessions on important determinants of weight loss maintenance, including developing skills to identify and manage high-risk situations for lapses, creating new helpful habits, breaking old unhelpful habits, and learning to manage interpersonal relationships and their impact on weight management.

Participants will receive 4 scheduled 30-minute telephone calls from a coach over the course of the intervention (after sessions 1, 3, 8, and 14), with an increasing amount of time between telephone calls as the intervention progresses to encourage autonomous motivation. Verbal contracting will occur at the end of the first coach call, where the participant commits to completing the intervention and what it involves. The remaining 3 coach calls will focus on reviewing exercises, troubleshooting, and transition planning (particularly for the gradual withdrawal of coach support). Participants may have up to 3 additional telephone calls with the coach if they require further support over the course of the intervention. The ethos for the coach support is that the participant is the *expert*, with the coach facilitating them to take ownership of their weight management by creating a collaborative relationship, drawing from the principles of motivational interviewing [41].

**Figure 3:**
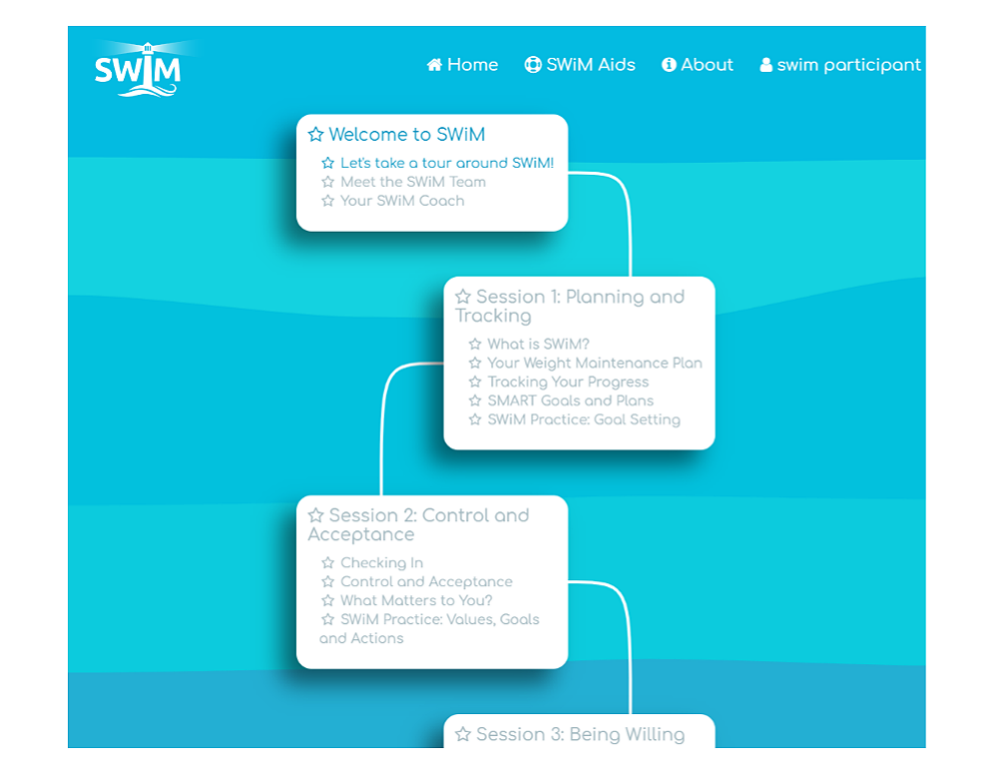
Screenshot of the SWiM (Supporting Weight Management) website showing the journey tracker. SMART: Specific Measured Active Realistic Time limited.

**Figure 4:**
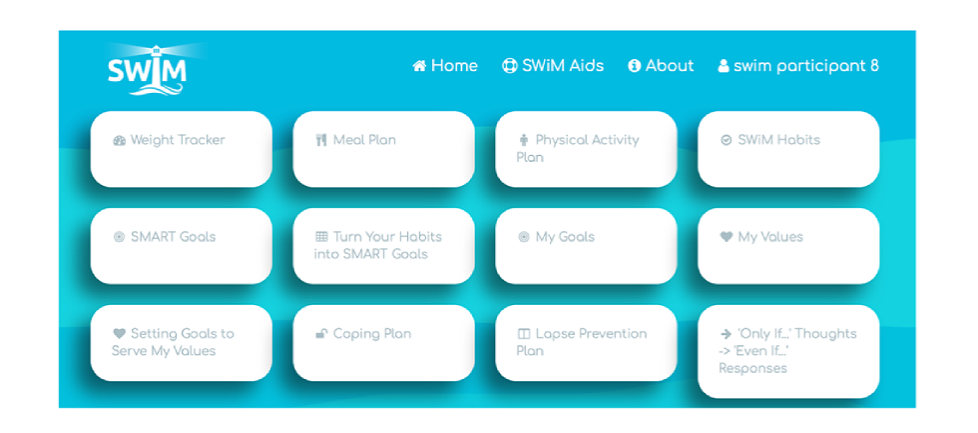
Screenshot of the SWiM (Supporting Weight Management) Aids tab. SMART: Specific Measured Active Realistic Time limited.

**Figure 5:**
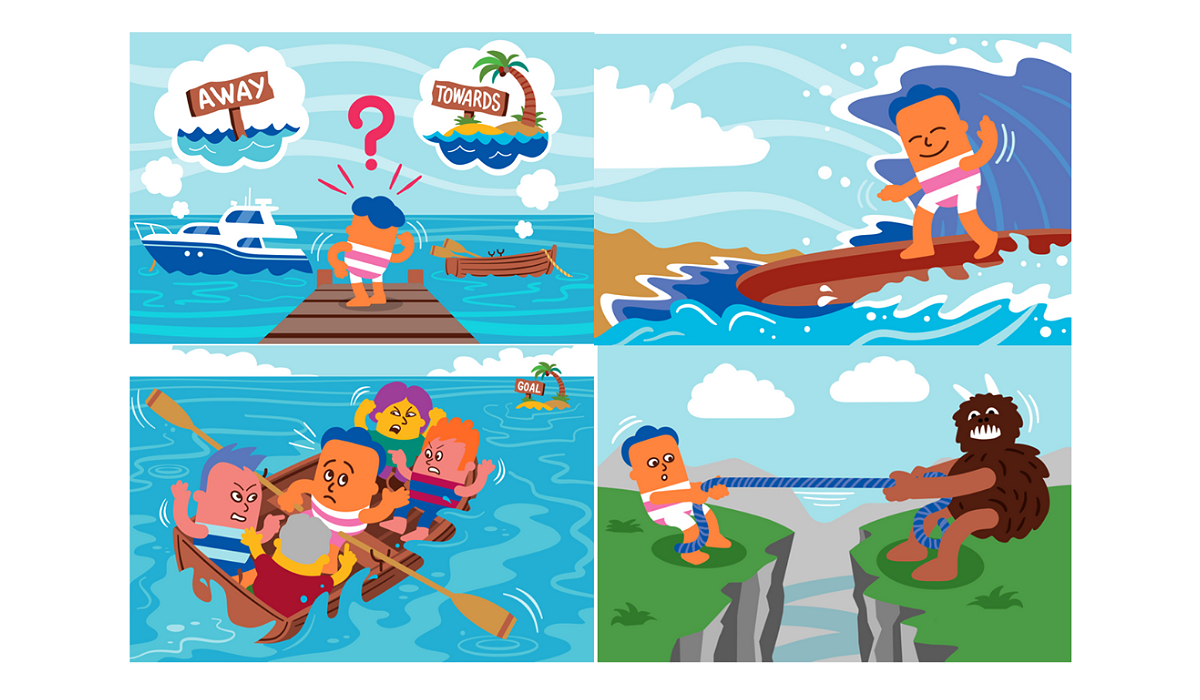
Examples of the graphic images created for SWiM (Supporting Weight Management).

The website platform was designed to be simple and interactive. Intervention content is divided into *SWiM Sessions*, each of which is subdivided into sections with activities. Progress through the sessions is presented as a *journey* using a map-like graphic down the center of the home page, and star icons light up when sessions (and subsections) are completed (Figure 3). Sessions can be exited at any point, and participants can easily return to the place they exited. Sessions and subsections are unlocked when the previous session or subsection is completed. Reflective exercises and behavioral experiments related to core skills and strategies are saved in a separate section of the website, called *SWiM Aids*, for easy access and use (Figure 4). The website also includes a visual weight tracker, which tracks weekly weight over time. The weight tracker generates a line graph as data are inputted by the participant and automatically sets a weight loss maintenance target *buffer* range with boundaries of +3 kg to −3 kg within which participants are encouraged to remain. The buffer range is adjustable as their weight changes; for example, if they continue to lose more weight or if they have regained weight and are finding it discouraging. Each session contains several cartoon-style images to illustrate the important concepts and metaphors drawn from ACT (Figure 5). Finally, session 13 includes an audio-video feature on self-acceptance.

Behavioral analysis revealed that all 3 components of the COM-B model (capability, opportunity, and motivation) will be targeted by the intervention to facilitate long-term weight management. The TDF was used to further elaborate on the COM-B components. Multimedia Appendix 4 outlines how each of the 3 components of the Behavior Change Wheel, their subcomponents, and corresponding TDF domains map onto elements of the intervention, with examples outlined. A total of 5 intervention functions and 27 BCTs were identified. Multimedia Appendix 5 outlines the intervention functions and their definitions, and indicates which program components correspond to each function. Multimedia Appendix 6 includes the BCTs included in the intervention and examples of how each is implemented.

## Discussion

### Principal Findings

The long-term impact and cost-effectiveness of weight management programs depend on posttreatment weight loss maintenance [7]. There is growing evidence that interventions based on 3wCBT, particularly ACT, could improve long-term weight management; however, these interventions are typically delivered face-to-face by psychologists, which limits the scalability of these types of interventions [9,12,42,43]. This paper describes an evidence-, theory-, and person-based approach to the development of an ACT-based intervention to support adults with overweight and obesity to maintain their weight loss in the long term. A key aim of the development was to design an intervention that could be delivered using digital technology and nonspecialists to minimize the resources needed for delivery at scale.

By drawing on a series of robust evidence syntheses [24-27], including a network meta-analysis conducted specifically for this project [9], we ensured that our intervention was informed by the latest scientific research on the determinants of weight loss maintenance, the behavioral interventions that are most effective for long-term weight management, and the experiences of people who have attempted to maintain weight loss. In-depth qualitative research provided additional insights into the specific needs of this target population, particularly the cognitive and behavioral strategies used by individuals who maintain their weight loss [28]. In addition, by modeling a justifiable cost for a weight loss maintenance program based on a hypothesized effect size, we were able to design an intervention within a set of specific resource parameters and increase the probability of the intervention being cost-effective.

We used the person-based approach to develop clear guiding principles based on collated evidence, including key intervention objectives and design features [23]. We then undertook an iterative cycle of intervention design and user testing to refine the content, design, and function. This facilitates the development of an intervention that is engaging for and relevant to target users. The involvement of a target user panel in the review and refinement of each intervention iteration helped ensure that target user feedback was given full consideration. Regular meetings with a stakeholder panel also ensured that any changes were considered within the context of existing care pathways and the experiences of health care practitioners and weight management service commissioners.

We have developed all the materials needed to deliver the web-based, guided self-help, ACT-based intervention, including the website platform, coach manual, and associated coach training materials. We have also finalized the logic model that specifies the hypothesized mechanisms of change of the intervention. In line with the MRC framework for the development of complex interventions in health care [21], the next phase for the development of the SWiM intervention will involve conducting a mixed methods feasibility study to evaluate the acceptability of the intervention from the perspectives of participants and the coaches who deliver the intervention and the feasibility of the intervention and study, including testing procedures, estimating recruitment and retention, and determining sample size for a randomized controlled trial. A protocol for a feasibility study was completed, and received ethical approval (ISRCTN12685964; March 5, 2021), with participant recruitment anticipated to begin in June 2021.

The systematic and theoretical development of the SWiM intervention outlined in this paper builds on the current evidence base of advanced intervention development methodology for digital weight management interventions [44-46]. By using this systematic, evidence- and theory-based development process, including the formal coding of BCTs and identification of hypothesized mechanisms of change [38,40], this emerging body of cutting-edge research will help us move forward the investigation of which BCTs work for whom, in which contexts, and delivered by what means, for effective long-term weight management [47].

### Conclusions

This paper highlights how an evidence-, theory-, and person-based approach can be applied to the development of a complex intervention to support weight loss maintenance for adults with overweight and obesity. The integrated, comprehensive, and iterative approach has facilitated the development of an intervention that is based on scientific theory and evidence but grounded in the experiences of the target users, stakeholders, and the context in which the intervention is intended to be delivered. Future intervention refinement will be guided by the findings of the planned feasibility study, which will evaluate the acceptability and feasibility of the intervention, and will inform a future trial of clinical and cost-effectiveness.

## Appendix

Multimedia Appendix 1 Guiding principles for the intervention.

Multimedia Appendix 2 The SWiM (Supporting Weight Management) intervention structure and content.

Multimedia Appendix 3 Outline of how the SWiM (Supporting Weight Management) intervention addresses each of the core acceptance and commitment therapy processes.

Multimedia Appendix 4 The COM-B model components targeted by the SWiM (Supporting Weight Management) intervention.

Multimedia Appendix 5 Outline of the SWiM (Supporting Weight Management) intervention components and corresponding intervention functions.

Multimedia Appendix 6 Behaviour change techniques included in the SWiM (Supporting Weight Management) intervention, numbered according to the BCTTV1.

## Abbreviations

3wCBT: third-wave cognitive behavioral therapies
ACT: acceptance and commitment therapy
BCT: behavior change technique
BWMP: behavioral weight management program
ICER: incremental cost-effectiveness ratio
IMP: Intervention Mapping Protocol
MRC: Medical Research Council
NHS: National Health Service
QALY: quality-adjusted life year
SBT: standard behavioral therapy
SWiM: Supporting Weight Management
TDF: Theoretical Domains Framework
WRAP: Weight loss program Referrals for Adults in Primary care
