# Supplementary material for: Development of a Web-Based, Guided Self-help, Acceptance and Commitment Therapy–Based Intervention for Weight Loss Maintenance: Evidence-, Theory-, and Person-Based Approach
Source: JMIR Form Res. 2022 Jan 7;6(1):e31801. doi: 10.2196/31801 (PMC8783282; doi:10.2196/31801)
Supplement: Multimedia Appendix 1 [file formative_v6i1e31801_app1.docx]

| Intervention design objectives  The intervention design objectives and features of the guiding principles for the Supporting Weight Management (SWiM) intervention. | Intervention features | Evidence (source) |
| --- | --- | --- |
| 1. To deliver an effective ACT-based intervention that can be delivered remotely and at scale at a cost of approximately £100 (US $132.39) per participant. | - Self-help program delivered via a web-based platform. - Intervention content to be underpinned by core principles and key skills of ACT^a^. - Include telephone support from a coach (guide) to enhance engagement and understanding of intervention content. - Nonspecialist personnel recruited for coach role to be trained for delivering the intervention using semistructured scripts (ie, a call center approach). | - Large target population but limited funding for programs (stakeholder panel). - Low capacity, high turnover, and/or lack of training of the existing health care staff to deliver support (stakeholder panel). - 3wCBT^b^ interventions shown to be more effective than standard behavioral therapy for weight management up to 24 months, with ACT-based interventions showing the most consistent evidence of effectiveness [9] (primary research). - Intervention costing approximately £100 (US $132.39) likely to be cost-effective (primary research). - eHealth versions of 3wCBT have been effective in other contexts [19], and early trials show promise for weight management (existing literature). - Digital interventions more effective with telephone support [31] (existing literature). - Cognitive behavioral therapy for weight management can be delivered by trained nonspecialists [14,15] (existing literature). - Participants desire follow-up support after weight-loss programs and find that in person support motivates program attendance and adherence [28] (primary research; target user panel). |
| 2. To build on participants’ existing knowledge and experience of weight management and what works for them. | - The intervention will be diet agnostic, meaning that participants can follow any diet of their choosing. - The program will start by asking participants to reflect on their experience and develop a personalized weight-loss maintenance plan. - The program will include opportunities to reflect on previous experience and identify what works for them and what their personal challenges are. - Each program session will also include reflective exercises and behavioral experiments. | - Target users have experience of losing weight and already have some knowledge of what works for them (target user panel; primary research). - Participants often have deeply ingrained beliefs about the diet that helped them to lose weight and the type of diet they can sustain long-term (target user panel; primary research). - Maintainers understand the different influences on their weight-management behaviors and learn from positive and negative experiences, including lapses [24,28] (primary research; existing literature). |
| 3. To encourage participants to take ownership of their weight management for the long term. | - Focus of the program will be on learning new strategies to support weight-management behaviors chosen by the participant rather than dictating the behaviors. - Coach support will consist of 4 scheduled telephone calls, with increasing time between sessions as the intervention progresses. - Participants can have 3 additional optional telephone calls with their coach if desired - A focus of the coach support will be transition to self-management. - Participants will have indefinite access to the program for future use when needed. | - Indefinite support is desired for weight-loss maintenance, but most participants will not continue to attend a program indefinitely (primary research; target user panel). - Reliance on program leader as a primary source of motivation makes weight-loss maintenance more difficult after the program ends [28] (primary research; existing literature; target user panel). - Support needs to be sustainable, and indefinite support is unrealistic and unaffordable (stakeholder panel). - Intrinsic motivation for weight-loss maintenance is associated with improved long-term weight-loss maintenance compared with external sources of motivation [24] (existing literature) |
| 4. To support participants in planning to navigate factors that commonly derail weight-loss maintenance, such as high-risk social and old unhelpful habits. | - The program will start by reflecting on experience and developing a personalized maintenance plan. - Include strategies for identifying and managing their own high-risk situations. - Include a specific module on managing interpersonal relationships. - Include a specific module on making new healthy habits and breaking old unhelpful habits. | - A key difference between maintainers and regainers is having a plan to continue with weight management strategies [28] (primary research; target user panel). - Social events and holidays are particularly challenging [28] (primary research; target user panel). - Maintainers anticipate potential lapses and have plans to manage these [28] (primary research; target user panel). - Regainers report difficulty managing relationships with family, friends, and colleagues around food criticisms, expectations, and social norms [28] (primary research; target user panel). - Turning weight-management behaviors into habits can help sustain them in the long term [24] (existing literature). |

^a^ACT: acceptance and commitment therapy.

^b^ 3wCBT: third-wave cognitive behavioral therapy.
