# Supplementary material for: Development of a Web-Based, Guided Self-help, Acceptance and Commitment Therapy–Based Intervention for Weight Loss Maintenance: Evidence-, Theory-, and Person-Based Approach
Source: JMIR Form Res. 2022 Jan 7;6(1):e31801. doi: 10.2196/31801 (PMC8783282; doi:10.2196/31801)
Supplement: Multimedia Appendix 2 [file formative_v6i1e31801_app2.docx]

| Week  Outline of the final Supporting Weight Management (SWiM) intervention content and structure. | SWiM Session | Content | Coach support |
| --- | --- | --- | --- |
| 1 | Welcome to SWiM | Let’s take a look around SWiM! |  |
|  |  | Meet the SWiM team |  |
|  |  | Your SWiM Coach |  |
| 1 | Session 1: Planning and Tracking | 1. What is SWiM? | 30 minute scheduled telephone call following completion of SWiM Session 1. |
|  |  | - 1. Your Weight Maintenance Plan |  |
|  |  | 1.2 Tracking Your Progress |  |
|  |  | 1.3 SMART Goals and Plans |  |
|  |  | 1.4 SWiM Practice: Goal Setting |  |
| 2 | Session 2: Control and Acceptance | 2.0 Checking in |  |
|  |  | 2.1 Control and Acceptance |  |
|  |  | 2.2 What Matters to You? |  |
|  |  | 2.3 SWiM Practice: Values, Goals and Actions |  |
| 3 | Session 3: Being Willing | 3.0 Checking in | 30 minute scheduled telephone call following completion of SWiM Session 3. |
|  |  | 3.1 Values and Goals |  |
|  |  | 3.2 Being Willing |  |
|  |  | 3.3 SWiM Practice: ‘Even If…’ Thoughts |  |
| 4 | Session 4: Overcoming Obstacles | 4.0 Checking in |  |
|  |  | 4.1 Identifying Your Obstacles |  |
|  |  | 4.2 Planning for Obstacles |  |
|  |  | 4.3 SWiM Practice: Being BOLD |  |
| 5 | Session 5: Being Active and Willing | 5.0 Checking in |  |
|  |  | 5.1 Physical Activity Recommendations |  |
|  |  | 5.2 Obstacles to Being Active |  |
|  |  | 5.3 Applying Willingness to Physical Activity |  |
|  |  | 5.4 SWiM Practice: Your Physical Activity Plan |  |
| 6 | Session 6: Emotional Eating | 6.0 Checking in |  |
|  |  | 6.1 What is Emotional Eating? |  |
|  |  | 6.2 Breaking the Cycle |  |
|  |  | 6.3 SWiM Practice: Emotional Responses Diary |  |
| 7 | Session 7: Stress Management | 7.0 Checking in |  |
|  |  | 7.1 Stress and Weight Gain |  |
|  |  | 7.2 Control What You Can, Accept What You Can’t |  |
|  |  | 7.3 Defusion: Unplugging the Dink |  |
|  |  | 7.4 Mindful Breathing |  |
|  |  | 7.5 SWiM Practice: Practising Defusion |  |
| 8 | Session 8: Forming Helpful Habits | 8.0 Checking in | 30 minute scheduled telephone call following completion of SWiM Session 8. |
|  |  | 8.1 Recap of Sessions 1 to 7 |  |
|  |  | 8.1 Forming Helpful Habits |  |
|  |  | 8.2 SWiM Practice: Forming Your New Habit |  |
| 9 | Session 9: Breaking Unhelpful Habits | 9.0 Checking in |  |
|  |  | 9.1 Breaking Unhelpful Habits |  |
|  |  | 9.2 Being Flexible |  |
|  |  | 9.3 SWiM practice: Breaking Your Unhelpful Habits |  |
| 10 | Session 10: Urges and Cravings | 10.0 Checking in |  |
|  |  | 10.1 We All Have Urges and Cravings |  |
|  |  | 10.2 A Recap of Defusion |  |
|  |  | 10.3 Urge Surfing |  |
|  |  | 10.4 SWiM Practice: Learning to Surf |  |
| 11 | Session 11: The Power of Sleep | 11.0 Checking in |  |
|  |  | 11.1 The Power of Sleep |  |
|  |  | 11.2 Sleep and Weight Management |  |
|  |  | 11.3 How to Get a Good Nights Sleep |  |
|  |  | 11.4 SWiM Practice: Forming Helpful Sleep Habits |  |
| 12 | Session 12: Friends and Family | 12.0 Checking in |  |
|  |  | 12.1 Friends and Family |  |
|  |  | 12.2 How to Get the Support You Need |  |
|  |  | 12.3 Breaking Unhelpful Food Rules |  |
|  |  | 12.4 SWiM Practice: Rule Breaking and Being Assertive |  |
| 13  [4-week break to practice skills] | Session 13: Weight Stigma and Body Image | 13.0 Checking in |  |
|  |  | 13.1 Weight Stigma |  |
|  |  | 13.2 How to Deal with Weight Stigma |  |
|  |  | 13.3 Body Image |  |
|  |  | 13.4 Self-Acceptance |  |
|  |  | 13.5 Physical Activity and Body Image |  |
|  |  | 13.6 SWiM Practice: Practicing Self-Acceptance |  |
| 18 | Session 14: Lapses and motivation | 14.0 Checking in | 30 minute scheduled telephone call following completion of SWiM Session 14. |
|  |  | 14.1 Lapse Versus Relapse |  |
|  |  | 14.2 Strategies to Prevent a Relapse |  |
|  |  | 14.3 Reversing Small Weight Gains |  |
|  |  | 14.4 Maintaining Motivation |  |
|  |  | 14.5 Going Forward |  |
