# Supplementary material for: Development of a Web-Based, Guided Self-help, Acceptance and Commitment Therapy–Based Intervention for Weight Loss Maintenance: Evidence-, Theory-, and Person-Based Approach
Source: JMIR Form Res. 2022 Jan 7;6(1):e31801. doi: 10.2196/31801 (PMC8783282; doi:10.2196/31801)
Supplement: Multimedia Appendix 3 [file formative_v6i1e31801_app3.docx]

| Core ACT processes  Outline of how the Supporting Weight Management (SWiM) intervention addresses each of the core ACT processes. | Supporting Weight Management (SWiM) program components |
| --- | --- |
| Acceptance | The concept of acceptance is introduced as an alternative to experiential avoidance of uncomfortable weight-management related internal (thoughts, feelings and sensations) and external (obstacles, such as social situations) experiences. Acceptance is introduced using a “tug of war” metaphor (Session 2: Control and Acceptance). Participants are supported to develop their acceptance skills by learning the concept of a ‘choice-point’, and completing cognitive exercises and behavioural experiments, such as turning “Only if” thoughts to “Even If” responses (Session 3: Being Willing). The process of acceptance is central to all sessions throughout the program, including sessions on physical activity, emotional eating and stress management. Specifically, participants are taught the skill of accepting some discomfort or less pleasure (for example, during exercise, experiencing hunger or cravings), in order to increase values-based action towards weight-management goals. |
| Cognitive defusion | The skill of cognitive defusion is taught to help participants to unhook from unhelpful thoughts and feelings that may derail their weight management. For example, cognitive defusion is taught as a skill to manage cravings and stress, prevent lapses from turning in to relapses, and foster self-acceptance. Cognitive defusion is used to help participants to distance themselves from unhelpful internal experiences and as a result, facilitate values-based action in line with their weight management goals. Metaphors and exercises such as “urge surfing” are used throughout the program to develop participants’ cognitive defusion skills. |
| Present-moment awareness | Participants are taught strategies to develop their present moment awareness throughout the SWiM program to facilitate psychological flexibility in their behaviours. Present moment awareness supports the management of internal (e.g. cravings, urges, low mood) and external (e.g. the influence of family and friends) experiences that may derail weight management, by faciitating non-judgemental, on-going awareness of thoughts, feelings or other private events, which allows participants to act more flexibly in response, in line with their weight management goals. Mindful breathing exercises and behavioural experiments, such as choosing a “BOLD” response to daily choice-points (i.e. **B**reathe deeply, slow down; **O**bserve what you are doing, feeling, and thinking; **L**isten to your values. Right now, what kind of person do you want to be?; **D**ecide on actions. Be BOLD and choose actions that move you towards your values), are included throughout the program. |
| Self as context | Self as context, i.e. “the observing self” is fostered throughout the SWiM program by teaching participants to step back and notice their own thinking and experiences of physical or emotional sensations (e.g. cravings and urges, emotions). This perspective-taking is facilitated by a range of exercises, such mindful breathing, defusion exercises, metaphors, and behavioural experiments (e.g. such as “BOLD”, described above). These exercises and experiments help to develop participants’ understanding that they can be aware of, or notice, their own internal and external experiences without fusing to them, which in turn, fosters their development of acceptance and defusion skills. |
| Values | The SWiM program fosters values clarification from the start to help participants to identify why weight management is important to them and how it will help them to live a more meaningful, values-based life. Furthermore, values clarification supports participants’ motivation to manage their weight throughout and beyond the SWiM program. A lighthouse metaphor is used to explain the concept of values, and exercises (such as linking their values to specific weight management goals) and behavioural experiments (such as practising willingness to take values-based action when faced with a choice-point) help participants to develop an awareness of their weight-management related values throughout the program. |
| Committed action | Once participants have clarified their values, developed goals that are guided by those values, and practiced taking values-based action throughout the program, the last session of the program introduces the idea of translating their values into committed action. This means engaging in larger patterns of effective action over time, guided by their weight-management related values, particularly when obstacles crop up, and motivation waxes and wanes. Participants are provided with several strategies to facilitate committed action, such as reminding themselves of their values and how weight management will help them to serve them, posting visual reminders of their weight-management related values in their environment, and publicy sharing their values and goals with others. Participants are also asked to develop a plan for maintaining their motivation in the long term, which includes their important weight-management related behaviours, potential challenges that may crop up, and strategies that they plan to use to enhance their commitment and increase their motivation. |
