# Supplementary material for: Development of a Web-Based, Guided Self-help, Acceptance and Commitment Therapy–Based Intervention for Weight Loss Maintenance: Evidence-, Theory-, and Person-Based Approach
Source: JMIR Form Res. 2022 Jan 7;6(1):e31801. doi: 10.2196/31801 (PMC8783282; doi:10.2196/31801)
Supplement: Multimedia Appendix 4 [file formative_v6i1e31801_app4.docx]

The COM-B model components targeted by the Supporting Weight Management (SWiM) intervention.

| COM-B model component | Sub-component and definition | TDF domain | Examples |
| --- | --- | --- | --- |
| Capability | Physical capability:  Physical skill, strength or stamina. | Physical skills | - Be physically active. |
|  | Psychological capability:  Knowledge or psychological skills, strength or stamina to engage in the necessary mental processes. | Knowledge | - Develop knowledge and understanding of the core concepts of acceptance and commitment therapy, as applied to weight management. - Develop knowledge and understanding of the determinants of, and barriers, to weight management in the long term. |
|  |  | Cognitive and interpersonal skills | - Develop the psychological skills to identify and overcome barriers to weight management, such as psychological flexibility and cognitive defusion. - Develop interpersonal skills, such as assertiveness, to manage the impact of relationships on weight management. |
|  |  | Memory, attention and decision processes | - Improve in-the-moment decision-making processes; notice daily ‘choice points’ and make goal-directed and valued-based choices. |
|  |  | Behavioural regulation | - Develop self-regulation skills, such as self-monitoring, planning and problem-solving. |
| Opportunity | Physical opportunity:  Opportunity afforded by the environment involving time, resources, locations, cues, physical ‘affordance’. | Environmental context and resources | - Adapt physical environments to include prompts and reminders for weight management behaviours. |
|  | Social opportunity:  Opportunity afforded by interpersonal influences, social cues and cultural norms that influence the way that we think about things, e.g. the words and concepts that make up our language. | Social influences | - Manage interpersonal relationships with regards to their impact on weight management behaviours. - Manage cultural norms, or family norms such as ‘food rules’. - Manage impact of weight stigma. |
| Motivation | Reflective motivation:  Reflective processes involving plans (self-conscious intentions) and evaluation (beliefs about what is good and bad). | Social/professional role and identity | - Include weight management behaviours as part of a new identity. |
|  |  | Beliefs about capabilities | - Believe that weight management will require improved cognitive and behavioural skills. |
|  |  | Optimism | - Maintain positive attitude following a lapse in order to prevent relapses. |
|  |  | Intentions | - Make plans to enact weight management behaviours. - Conduct problem-solving to plan for and overcome obstacles to weight management. |
|  |  | Goals | - Set relevant weight management goals to facilitate values-based living. |
|  |  | Beliefs about consequences | - Believe that obesity leads to health consequences and impacts on relevant values and goals. |
|  | Automatic motivation**:**  Automatic processes involving emotional reactions, desires (wants and needs), impulses, inhibitions, drive states and reflex responses. | Reinforcement | - Create and reinforce new helpful habits and routines. - Break unhelpful habits that derail weight management. |
|  |  | Emotion | - Manage emotions and stress to prevent urges and cravings for calorie-dense food. |
