# Supplementary material for: Development of a Web-Based, Guided Self-help, Acceptance and Commitment Therapy–Based Intervention for Weight Loss Maintenance: Evidence-, Theory-, and Person-Based Approach
Source: JMIR Form Res. 2022 Jan 7;6(1):e31801. doi: 10.2196/31801 (PMC8783282; doi:10.2196/31801)
Supplement: Multimedia Appendix 5 [file formative_v6i1e31801_app5.docx]

Outline of the Supporting Weight Management (SWiM) intervention components and corresponding intervention functions.

| Intervention functions and definition | SWiM program components |
| --- | --- |
| Education:  Increasing knowledge or understanding. | The SWiM program aims to increase knowledge and understanding of the core concepts of acceptance and commitment therapy, as applied to weight management. It also aims to increase knowledge and understanding of the determinants of, and barriers to, successful weight loss maintenance, such as high-risk situations for lapses, habit formation and discontinuation, and interpersonal relationships. Additionally, the role of the coach is to facilitate participants’ understanding of the intervention content and their knowledge of ACT in application to their weight management. |
| Training:  Imparting skills. | Reflective exercises and behavioural experiments are a core part of each SWiM session and serve to impart the skills and strategies required for successful weight loss maintenance, such as planning, problem-solving, self-regulation and maintaining motivation. The role of the coach includes helping participants to develop such skills by facilitating their understanding of the exercises and reflection on their practice. |
| Environmental restructuring:  Changing the physical or social environment. | Some of the SWiM sessions encourage participants to change their physical environment to include prompts and cues for healthy behaviours (e.g. Session 8: Forming Helpful Habits; Session 14: Lapses and Maintaining Motivation), or change their social environment to increase social support for their weight management (e.g. Session 12: Family and Friends). |
| Modelling:  Providing an example for people to aspire to or imitate. | An animated character called “Chris” has been created by a graphic designer to illustrate the ACT metaphors used throughout the program. Chris provides an example for participants to aspire to. |
| Enablement:  Increasing means/reducing barriers to increase capability (beyond education and training) or opportunity (beyond environmental restructuring). | The SWiM program will provide participants with behavioural and cognitive support to manage their weight. The program goes beyond education and training as participants receive tailored support from a SWiM coach via four scheduled telephone calls over the course of the program (with a further three optional calls available if desired). |
