# Supplementary material for: Development of a Web-Based, Guided Self-help, Acceptance and Commitment Therapy–Based Intervention for Weight Loss Maintenance: Evidence-, Theory-, and Person-Based Approach
Source: JMIR Form Res. 2022 Jan 7;6(1):e31801. doi: 10.2196/31801 (PMC8783282; doi:10.2196/31801)
Supplement: Multimedia Appendix 6 [file formative_v6i1e31801_app6.docx]

Behaviour Change Techniques included in the Supporting Weight Management (SWiM) intervention, numbered according to the BCTTV1.

| Number and Label | Label | Definition | Examples |
| --- | --- | --- | --- |
|  | Goals and planning |  |  |
| 1.1 | Goal setting (behaviour) | Set or agree a goal defined in terms of the behaviour to be achieved. | - ‘Session 1: Planning and Tracking’ introduces goal-setting. - The SWiM Practice for Session 1 requires two specific goals to be set. This exercise is then stored in the SWiM Aids so that participants can set new goals as they progress through the program. |
| 1.2 | Problem solving | Analyse, or prompt the person to analyse, factors influencing the behaviour and generate or select strategies that include overcoming barriers and/or increasing facilitators. | - ‘Session 4: Overcoming Obstacles’ requires participants to identify their barriers to weight management behaviours and plan solutions to overcome them. |
| 1.4 | Action planning | Prompt detailed planning of performance of the behaviour (must include at least one of context, frequency, duration and intensity). Context may be environmental (physical or social) or internal (physical, emotional or cognitive). | - ‘Session 1: Planning and Tracking’ encourages participants to plan their meals (including breakfast, lunch, dinner and snacks) and ‘Session 5: Being Active and Willing’ encourages participants to plan their physical activity (including activity, duration, day and time). |
| 1.5 | Review behaviour goal(s) | Review behaviour goal(s) jointly with the person and consider modifying goal(s) or behaviour change strategy in light of achievement. This may lead to re-setting the same goal, a small change in that goal or setting a new goal instead of (or in addition to) the first, or no change. | - Goal-setting exercises encourage participants to later return to the website to reflect on their progress and record this information on the website. The SWiM coach may later review their progress with them on the telephone. Goals may be adjusted accordingly. |
| 1.6 | Discrepancy between current behaviour and goal | Draw attention to discrepancies between a person’s current behaviour (in terms of the form, frequency, duration or intensity of that behaviour) and the person’s previously set outcome goals, behavioural goals or action plans (goes beyond self-monitoring of behaviour). | - Goal-setting exercises encourage participants to later return to the website to reflect on their progress and record this information on the website. The SWiM coach may later review their progress with them on the telephone. Goals may be adjusted accordingly. Both of these exercises will highlight any discrepancies between their behaviours and goals. |
| 1.8 | Behavioural contract | Create a written specification of the behaviour to be performed, agreed by the person, and witnessed by another. | - At the start of the program, prior to session 1, participants are asked via the website to agree to a written contract that requires them to complete 4 telephone calls with their coach, and complete a SWiM session each, including the exercises and SWiM practices. Participants are asked to press an ‘I agree’ or ‘I disagree’ button to affirm their commitment to the SWiM program. - During the first coach call, the coach takes participants through the contract again over the phone and asks participants to reaffirm their commitment to the SWiM program. |
| 1.9 | Commitment | Ask the person to affirm or reaffirm statements indicating commitment to change the behaviour. | - During the first coach call, the coach takes participants through the contract again over the phone and asks participants to reaffirm their commitment to the SWiM program. |
|  | Feedback and monitoring |  |  |
| 2.1 | Monitoring of behaviour by others without feedback | Observe of record behaviour with the person’s knowledge as part of a behaviour change strategy. | - Participants are made aware that coaches will have access to their data entries and will review for the coaching sessions. |
| 2.4 | Self-monitoring of outcome(s) of behaviour | Establish a method for the person to monitor and record the **outcome(s)** of their behaviour as part of a behaviour change strategy. | - Participants are asked to record their weight in the weight tracker at the start of each session (weekly). |
|  | Social support |  |  |
| 3.1 | Social support (unspecified) | Advise on, arrange or provide social support (e.g. from friends, relatives, colleagues, buddies or staff) or non-contingent praise or reward for performance of the behaviour. It includes encouragement and counselling, but only when it is directed the **behaviour.** | - ‘Session 12: Family and Friends’ discusses the impact of interpersonal relationships on weight management behaviours. It helps participants to navigate such relationships so as to increase social support for weight management. - Participants are supported through the SWiM program by a coach. |
|  | Shaping knowledge |  |  |
| 4.1 | Instruction on how to perform a behaviour | Advise or agree on how to perform the behaviour. | - Participants are provided with skills training throughout the SWiM program. For example, they are instructed on how to practice willingness, perform cognitive defusion and practice self-acceptance. |
| 4.2 | Information about antecedents | Provide information about antecedents | - In ‘Session 6: Emotional Eating’, participants are asked to identify triggers for emotional eating (e.g. thoughts, feelings or situations). |
| 4.4 | Behavioural experiments | Advise on how to identify and test hypotheses about the behaviour, its causes and consequences, by collecting and interpreting data. | - Each SWiM session includes a behavioural experiment, called ‘SWiM Practice’, where participants test out the skills they have developed in each session. |
|  | Natural consequences |  |  |
| 5.1 | Information about health consequences | Provide information (e.g. written, verbal, visual) about health consequences of performing the behaviour. | - The program provides information on the health consequences of obesity and related behaviours such as physical inactivity and stress. |
| 5.4 | Monitoring of emotional consequences | Prompt assessment of feelings after attempts at performing the behaviour. | - ‘Session 6: Emotional Eating’ asks participants to reflect and write down how they feel after implementing an alternative behaviour, instead of emotional eating, in response to a trigger. |
| 5.5 | Anticipated regret | Induce or raise awareness of expectations of future regret about performance of the unwanted behaviour. | - ‘Session 6: Emotional Eating’ raises awareness of the regret and related negative emotions one can feel after emotional eating. |
| 5.6 | Information about emotional consequences | Provide information (e.g. written, verbal, visual) about emotional consequences of performing the behaviour. | - ‘Session 6: Emotional Eating’ highlights that choice alternative ‘activating’ or ‘soothing’ behaviours, instead of eating, in response to emotions, will help to make the participant feel better. |
| 7. | Associations |  |  |
| 7.1 | Prompts/cues | Introduce or define environmental or social stimulus with the purpose of prompting or cueing the behaviour. The prompt or cue would normally occur at the time or place of performance. | - ‘Session 8: Forming Helpful Habits’ introduces the concept of cues to explain habit formation and encourages participants to identify cues to build new, helpful habits. - ‘Session 14: Lapses and Maintaining Motivation’ asks participants to create visual prompts in their physical environment to remind them to stay focused on their weight management behaviours. - Participants receive automatic email reminders if they do not complete a session each week. |
| 8. | Repetition and substitution |  |  |
| 8.2 | Behavioural substitution | Prompt substitution of the unwanted behaviour with a wanted or neutral behaviour. | - Many sessions throughout the program encourage behavioural substitution of unwanted eating behaviour. For example, ‘Session 4: Overcoming Obstacles’ asks participants to plan an alternative behaviour (e.g. go for a walk) instead of an unwanted behaviour (e.g. emotional eating) in response to an obstacle (e.g. feel too tired to exercise). |
| 8.3 | Habit formation | Prompt rehearsal and repetition of the behaviour in the same context repeatedly so that the context elicits the behaviour. | - ‘Session 8: Forming Helpful Habits’ introduces the concept of habit formation and encourages participants to create new, helpful habits to support their weight management over the long term. |
| 8.4 | Habit reversal | Prompt rehearsal and repetition of an alternative behaviour to **replace** an unwanted habitual behaviour. | - ‘Session 9: Breaking Unwanted Habits’ introduces the concept of habit reversal by using psychological flexibility (e.g. encouraging the participant to go for a walk instead of watching television in response to a cue, such as coming home from work). |
| 8.7 | Graded tasks | Set easy-to-perform tasks, making them increasingly difficult, but achievable, until behaviour is performed. | - When setting goals, participants are encouraged to start small and build up gradually. For example, in ‘Session 5: Being Active and Willing’, participants are encouraged to plan their physical activity by setting small, achievable goals first, and then gradually increasing their physical activity as they progress. |
| 11. | Regulation |  |  |
| 11.2 | Reduce negative emotions | Advise on ways of reducing negative emotions to facilitate performance of the behaviour. | - Participants are advised on how to manage negative emotions throughout the program, using acceptance and commitment therapy. For example, ‘Session 7: Stress Management’ provides strategies for participants to manage stress, including the use of cognitive defusion. |
| 12. | Antecedents |  |  |
| 12.1 | Restructuring the environment | Change, or advise to change the physical environment in order to facilitate performance of the wanted behaviour or create barriers to the unwanted behaviour (other than prompts/cues, rewards and punishments). | - Participants might identify restructuring their physical environment as a strategy to overcome a barrier. For example, in ‘Session 4: Overcoming Obstacles’, participants might identify that having chocolate in the house for the children is an obstacle to their weight management and so they may alter their environment by throwing away the chocolate. |
| 12.4 | Distraction | Advise or arrange to use an alternative focus for attention to avoid triggers for unwanted behaviour. | - ‘Session 6: Emotional Eating’ encourages participants to implement alternative activities in response to triggers, instead of emotional eating. ‘Activating’ or ‘soothing’ activities are selected to distract from the desire to eat. |
| 13. | Identity |  |  |
| 13.1 | Framing/reframing | Suggest the deliberate adoption of a perspective or new perspective on behaviour (e.g. its purpose) in order to change cognitions or emotions about performing the behaviour. | - The SWiM program is based on acceptance and commitment therapy and so teaches participants a new perspective of acceptance of difficult thoughts, feelings or situations, and therefore tolerance of discomfort in order to reach their values-based goals. |
| 13.4 | Valued self-identity | Advise the person to write or complete rating scales about a cherished value or personal strength as a means of affirming the person’s identity as part of a behaviour change strategy. | - ‘Session 2: Control and Acceptance’ requires participants to identify their values and explicitly state how managing their weight will allow them to live a meaningful live based on these values. |
